# Research Progress and Technological Application Prospects of Comprehensive Evaluation Methods for Egg Freshness

**DOI:** 10.3390/foods14091507

**Published:** 2025-04-25

**Authors:** Zhouyang Gao, Jiangxia Zheng, Guiyun Xu

**Affiliations:** College of Animal Science and Technology, China Agricultural University, Beijing 100193, China; gaozhouyangcau@163.com

**Keywords:** egg freshness evaluation, non-destructive detection technologies, spectroscopic analysis, computer vision technology

## Abstract

Eggs, recognized as a “complete nutritional food”, constitute a crucial source of high-quality protein and maintain an essential position in China’s animal protein supply system. However, extended storage periods induce biochemical degradation, including protein denaturation, air cell expansion, and microbial growth, substantially affecting both food safety and nutritional value. As consumer demand for food quality assurance increases, research in egg freshness evaluation has made substantial progress. While existing studies have focused on isolated detection methods for egg freshness, there remains a critical gap in systematically integrating multidisciplinary approaches and evaluating their synergistic potential for comprehensive quality assessment. This review systematically categorizes conventional and emerging detection methodologies, including sensory assessment, physical characterization, chemical analysis, and intelligent detection technologies. The paper presents a comprehensive analysis of current research developments while offering perspectives on practical applications and future directions for egg freshness evaluation systems.

## 1. Introduction

Eggs, recognized as a nutrient-dense food, provide essential proteins, lipids, minerals, and vitamins [1]. Structurally composed of shell, yolk, and albumen, fresh eggs demonstrate specific compositional characteristics: yolks contain 47–48% water, 16% protein, 32–35% lipids, and 1% carbohydrates, while albumen comprises 89% water, 10% protein, and minimal lipids [2]. Their comprehensive nutritional profile establishes their global dietary significance as both a staple food and an essential ingredient in processed products. Eggs are a nutrient-dense food source, offering high-quality proteins with a PDCAAS score of 1.0 and containing all essential amino acids. Yolk lipids, primarily oleic acid and DHA, support cell membrane and neurological health, while yolks provide vital micronutrients, including fat-soluble vitamins and essential minerals. Although low in carbohydrates, they contain antiviral glycoproteins. Additionally, bioactive compounds such as lysozyme and ovotransferrin have applications in pharmaceuticals and functional foods, targeting health issues like metabolic syndrome and age-related disorders, and enhancing neuroprotection and immune function [2,3].

Global egg production in 2024 reached approximately 1.3 trillion eggs, with China dominating at 49% of total output (29.4 million tons), driven by large-scale farming and technological advancements. India exhibited significant growth, producing 142.7 billion eggs, supported by population expansion and low-cost feed. The U.S. production declined by 3.9% (76.8 billion eggs) due to avian influenza outbreaks and flock reductions. Brazil and the EU contributed 45.8 billion eggs (+4.8%) and 6.66 million tons (−1.83%), respectively, reflecting regional challenges like feed costs and animal welfare policies. Globally, per capita consumption averaged 162 eggs/year, with disparities between high-consumption regions [4]. However, the industry confronts significant challenges in quality preservation across the supply chain. Eggs, being perishable commodities, experience progressive freshness deterioration from farm to table, intensified by inadequate temperature and humidity controls during storage and transportation [5]. This degradation diminishes their economic value and increases food safety risks. Despite increased public awareness of food safety and nutritional health, China currently lacks standardized regulations for egg shelf life determination. Market practices depend on post-laying storage duration as a freshness indicator—an insufficient metric that neglects environmental variables or mechanical damage during handling, which facilitates bacterial contamination.

Freshness, the primary quality parameter, directly influences eggs’ edibility, nutritional integrity, and processing suitability [6]. Specifically, albumen proteins exhibit functional properties essential for food manufacturing, including foaming capacity and gelling properties, which deteriorate as freshness declines [7]. Additionally, decreased bioavailability of bioactive compounds and modified physicochemical characteristics of yolk/albumen compromise both safety and nutritional efficacy, causing substantial economic losses in the fresh egg industry.

Precise freshness evaluation is thus crucial for ensuring food safety and consumer health [8,9]. As consumer demand for food quality assurance increases, this review addresses this gap by categorizing conventional and emerging detection methodologies—including sensory assessment, physical characterization, chemical analysis, and intelligent detection technologies—and proposing a novel framework to bridge the divide between laboratory-based innovations and scalable industrial applications. We further highlight the underexplored role of multi-modal data fusion and machine learning in overcoming limitations of single-method approaches, which has been largely overlooked in the prior literature. This review systematically examines conventional and emerging assessment methodologies, including sensory–physical analyses, chemical–biological markers, and advanced, non-destructive detection technologies. Through a comparative analysis of their efficacy, limitations, and practical applicability, this work seeks to establish a comprehensive framework for stakeholders to enhance freshness determination in both commercial and research contexts.

## 2. Egg Freshness Evaluation Methodology

### 2.1. Mechanisms of Freshness Degradation

The reduction in egg freshness during storage primarily results from the synergistic interaction between continuous material exchange processes and microbial spoilage mechanisms. From the physicochemical perspective, dynamic material transport continues through the eggshell pores; extended storage enables continuous outward diffusion of moisture and carbon dioxide [10]. This trans-shell migration induces a significant pH elevation in the egg white, causing reduced internal pressure and progressive expansion of the air cell, evidenced by simultaneous increases in both diameter and height of this structure [11]. The alkaline shift in egg white pH accelerates two critical deterioration processes: the rapid thinning of the thick egg white (measured by decreasing Haugh unit values) and the establishment of an osmotic gradient driving water migration from albumen to yolk. This inter-compartmental fluid transfer causes yolk liquefaction and accelerates the decay of yolk index [12].

Regarding microbial involvement, contamination risks emerge from both indigenous microbiota and external microbial invasion. The metabolic activities of these microorganisms produce multiple detrimental effects [13,14,15]: proteolytic degradation of albumen nutrients accelerates protein thinning and Haugh unit reduction; metabolic byproducts alter osmotic equilibrium, exacerbating fluid migration to yolk; and volatile decomposition products (e.g., CO_2_ and water) facilitate component leakage. These microbial actions create a self-perpetuating deterioration cycle characterized by continuous pH elevation, air cell enlargement, and progressive quality degradation. The mutually reinforcing nature of these physicochemical and biological mechanisms ultimately results in systemic quality deterioration in stored eggs.

### 2.2. Traditional Sensory and Physical Detection Methods

During egg storage, sensory changes in freshness can be assessed through odor, taste, and structural appearance [16]. Sensory detection requires no specialized instruments or reagents, depending instead on inspectors’ expertise in visual inspection, auditory evaluation, tactile assessment, and olfactory examination. This method remains the most widely utilized and accessible approach for daily freshness evaluation due to its simplicity and cost-effectiveness.

Visual inspection evaluates eggshell cleanliness, integrity, and coloration. Eggshell colors vary by breed, primarily classified into four categories: white, brown, light brown, and green [17]. Fresh eggs display a rough, intact surface with a frost-like gelatinous membrane. Signs of aging include membrane separation, discoloration (grayish tones), or mold formation. Examination of cracked eggs reveals internal characteristics: fresh egg whites appear thick, transparent, and either colorless or faint yellow-green. As eggs age, the albumen becomes thinner, transparency decreases under light, and yolks darken with slight mobility, indicating reduced freshness [18]. Fresh eggs contain thick, elastic chalazae firmly securing the yolk, while deteriorated eggs exhibit thinning, detached, or absent chalazae [19].

Auditory evaluation involves shaking or tapping eggs. Fresh eggs remain silent when shaken, while spoiled or aged eggs produce watery sloshing sounds. Shell tapping produces distinct acoustic patterns: fresh eggs generate a solid “brick-like” resonance, cracked eggs create a dull rattle, and moldy or rotten eggs emit a brittle “ceramic tile” sound [20]. Tactile assessment examines surface texture and weight. Fresh eggs initially present a slightly rough texture that smoothens during storage. Mold-contaminated or adherent-shelled eggs develop a sticky surface. Fresh eggs demonstrate notably greater weight compared to aged specimens. Olfactory detection identifies aromas: fresh eggs remain odorless, while stale or spoiled eggs produce unpleasant or sulfurous odors [21].

Physical analyses encompass density and air cell measurements [22]. Egg density inversely relates to freshness due to moisture loss through porous shells during storage, diminishing mass and buoyancy. Saltwater flotation tests utilize this principle: fresher eggs sink in denser solutions, while older ones float. Air cell size, affected by storage humidity and egg quality, functions as another freshness indicator [23]. National standards specify maximum air cell heights for graded eggs: premium (≤4 mm), first-grade (≤6 mm), second-grade (≤8 mm), and third-grade (≤9.5 mm). Measurement requires backlighting eggs on transparent, calibrated plates to observe air cell boundaries through the translucent shell [24].

Although cost-effective for household or small-scale applications, traditional methods present limitations. Subjectivity in sensory evaluation, insensitivity to early biochemical deterioration (e.g., protein denaturation), potential sample damage during testing, and inefficiency in high-throughput industrial applications have necessitated the adoption of advanced techniques such as spectroscopic analysis and electronic nose systems in modern poultry industries.

### 2.3. Physicochemical Indicator Detection Methods

During egg storage, physicochemical indicators of freshness experience systematic changes, including weight loss, moisture migration between the egg white and yolk, increased egg white pH, and liquefaction of the albumen [25]. These changes primarily result from water and carbon dioxide volatilization from the egg white, combined with ovomucin protein hydrolysis, which reduces the viscosity and thickness of the thick albumen layer. Based on these mechanisms, researchers have established multiple freshness evaluation metrics, particularly the Haugh unit (HU), yolk index, and egg white pH.

#### 2.3.1. Egg White Physicochemical Indicators

Egg white proteins manifest in two morphological states: thin albumen and thick albumen. During storage, the thick albumen gradually transforms due to protein hydrolysis [26]. When a fresh egg’s contents are deposited onto a flat glass surface, the thick albumen forms a cohesive layer surrounding the yolk. With time, protein degradation causes the thick albumen to thin, reducing its height and subsequently decreasing the Haugh unit value [27]. The Haugh unit, an internationally recognized freshness parameter standardized by the U.S. Department of Agriculture (USDA), measures freshness through a formula incorporating egg weight and thick albumen height. Higher HU values indicate better freshness, with eggs classified into three quality grades based on HU ranges (Table 1). This metric effectively measures the structural integrity of the thick albumen, functioning as a sensitive indicator of proteolytic changes during storage [28].

The pH of egg white is controlled by dissolved CO_2_, carbonate/bicarbonate ions, and protein interactions [29]. Fresh egg white demonstrates a pH range of 7.6–7.9. During storage, CO_2_ escapes through the porous shell, gradually increasing the pH to 9.0–9.7. The most significant pH increase occurs within the first three days post-laying, reaching approximately 9.0 due to accelerated CO_2_ dissipation [30]. This pH shift serves as a reliable freshness indicator, measurable via pH meters.

Protein degradation through enzymatic and microbial activity produces alkaline volatile compounds, primarily ammonia and amines, collectively known as Total Volatile Basic Nitrogen (TVBN). Increased TVBN levels correlate directly with egg aging [31]. Quantitative analysis typically utilizes semi-micro distillation methods using Kjeldahl apparatus to isolate and measure these nitrogenous compounds.

Recent studies identify additional freshness indicators in egg white. Changes in compounds such as uridine and pyroglutamic acid during storage demonstrate potential for non-invasive freshness evaluation [32]. Specifically, furosine—a product of Amadori compound hydrolysis—and ε-N-(2-furoylmethyl-L-lysine) show particular promise [33]. These markers demonstrate high reproducibility in fresh eggs and remain stable regardless of egg mass, hen age, or storage humidity. Research confirms furosine’s effectiveness as a reference metric for assessing shell egg freshness, providing a robust alternative to conventional parameters [34].

#### 2.3.2. Yolk Physicochemical Indicators

Surrounded by a semipermeable membrane, the yolk undergoes osmotic interactions with the egg white due to its higher salt concentration. This gradient facilitates salt diffusion into the albumen and water movement into the yolk, modifying yolk structure [35]. The yolk index—calculated as yolk height (mm) divided by yolk diameter (mm)—measures these changes. Fresh yolks present a hemispherical profile with high index values (Table 2), while storage-induced hydrolysis of lipids and proteins decreases viscosity, resulting in yolk flattening and index reduction [36]. Assessment requires egg breakage and vernier caliper measurement of yolk dimensions. Although simple and economical, this destructive method limits application to laboratory analyses or production-line sampling, preventing non-destructive quality monitoring.

The yolk membrane (a multilayered protein structure surrounding the yolk), functions as a freshness indicator, with its mechanical strength indicating resistance to external forces [37]. Commonly measured using a texture analyzer, this method utilizes a stainless-steel probe to compress the membrane vertically at the yolk’s apex, measuring rupture force, deformation degree, and work performed during penetration. Akarca et al. established that surface coatings enhance yolk membrane strength in long-stored eggs, correlating with freshness predictions from Haugh units and albumen pH [38]. This correlation validates texture analysis as a complementary freshness assessment tool.

### 2.4. Comparative Analysis of Traditional Detection Methods

Different freshness indicators represent distinct aspects of egg quality changes during storage, with comparative outcomes of traditional methods summarized in Table 3 and Table 4. Sensory evaluation depends significantly on experienced personnel but exhibits subjectivity and inefficiency, exacerbated by increasing labor costs. Physicochemical analysis, particularly through Haugh units and yolk indices, evaluates parameters such as albumen height, yolk dimensions, and air cell size. Traditionally, higher Haugh units and yolk index values signify superior freshness [39].

However, emerging anomalies challenge this established understanding. Market reports of “rubber eggs” reveal yolks maintaining unusual elasticity and elevated yolk indices despite extended storage, contradicting conventional freshness criteria [40]. Zhang et al. demonstrated that dietary supplementation with 1.5% oxidized soybean oil in hens produced eggs with significantly higher Haugh units compared to corn–soybean meal controls (*p* < 0.05), reflecting the atypical Haugh unit persistence observed in “rubber eggs” [41]. These findings reveal limitations in depending solely on physicochemical metrics for freshness assessment. Furthermore, traditional methods necessarily require egg destruction for measurement, restricting their utility to laboratory sampling or small-scale quality checks. Such approaches prove inadequate for modern industrial requirements of rapid, non-destructive, and high-throughput detection systems. This methodological limitation emphasizes the necessity for incorporating advanced analytical technologies into egg quality evaluation protocols.

### 2.5. Modern Non-Destructive Detection Technologies and Integrated Models

Propelled by rapid advancements in information and computer technologies, non-destructive detection systems—particularly computer vision—have emerged in smart agriculture for their cost-effectiveness, high accuracy, and rapid assessment capabilities, establishing foundations for their application in egg freshness evaluation. Current non-destructive techniques encompass infrared spectroscopy, hyperspectral imaging, electronic nose systems, and machine vision, among others. These technologies facilitate comprehensive analysis of internal and external egg quality parameters without compromising sample integrity, providing scalable solutions for industrial quality control.

#### 2.5.1. Electronic Nose Detection

Electronic nose (e-nose) technology utilizes sensor arrays composed of multiple gas-sensitive elements, each selectively responsive to specific volatile compounds [42]. By replicating human olfaction, it analyzes odor profiles generated during egg freshness degradation [43]. As eggs age, volatile compounds including hydrogen sulfide, carbon dioxide, and organic acids escape through the shell pores, modifying gas composition [44]. The sensor array detects these changes, transforms them into electrical signals, and processes the data through pattern recognition algorithms to evaluate freshness.

Recent studies have demonstrated substantial advancements in e-nose applications for egg quality evaluation. Yi et al. employed principal component analysis (PCA) to reduce dimensionality of volatile compound data, achieving cumulative variance interpretation rates of 95.70% and 93.71%, effectively differentiating eggs across storage periods [45]. Studies integrated e-nose data with Haugh units (HU) and yolk indices, establishing multivariate linear regression (MLR) models with prediction coefficients (Rp) of 0.87 (HU) and 0.84 (yolk index) [46]. Rocha et al. developed genetically optimized neural network models for storage duration classification (accuracy > 90%) and quadratic regression models (Rp > 0.9) for HU and yolk index prediction [47]. Ji’s self-developed e-nose system combined with random subspace ensemble learning achieved 88.57% accuracy in storage day classification [48]. Wang et al. constructed quadratic polynomial stepwise regression models under varying storage conditions, yielding R^2^ values of 0.91 (HU) and 0.93 (yolk index) [49]. Yu utilized quartz crystal microbalance sensors to predict shelf life (R^2^ = 0.96) [50], while Li et al. implemented wavelet energy features in SVM models for yolk index prediction (mean relative error: 7.56%) [51].

Although widely adopted in food processing, agriculture, and healthcare for its efficiency and simplicity, e-nose technology faces several limitations [52]. Environmental factors such as temperature, humidity, and farming practices may interfere with volatile compound detection. Furthermore, the technique exclusively addresses chemical indicators, lacking capability to identify physical defects such as shell cracks [53]. Accurate discrimination of overlapping volatile signatures remains a significant technical challenge, particularly during advanced storage stages when multiple degradation products coexist. These constraints highlight the necessity for complementary methods to enhance reliability in industrial freshness assessment systems.

#### 2.5.2. Spectral Analysis Technology

Spectral analysis technology represents a non-destructive detection method that utilizes the absorption, scattering, or emission characteristics of materials at specific wavelengths to provide information on chemical composition, structural features, and physical properties [54]. In egg freshness evaluation, it functions based on light interaction principles (transmission, refraction, reflection), analyzing optical phenomena during light penetration to establish mathematical models for quality assessment [55]. Various spectral techniques—including near-infrared (NIR), Raman, and hyperspectral imaging—serve essential roles in revealing freshness-related changes. The selection of chemometric methods directly impacts model robustness and interpretability. While unsupervised methods (e.g., PCA) efficiently reduce spectral dimensionality, supervised techniques (e.g., PLSR and SVR) enable targeted mapping of spectral features to freshness indicators. Recent advancements in hybrid optimization (GA-BPNN) and ensemble learning (LS-SVM) further address nonlinearity and noise challenges, as summarized in Table 5.

Investigators have extensively explored NIR applications for egg quality monitoring. Yang et al. integrated hyperspectral images with spectral data using parallel and progressive fusion techniques, demonstrating effective freshness identification, with progressive fusion exhibiting superior feature recognition [56]. Abdanan et al. achieved 94% freshness classification accuracy using fast Fourier transform (FFT) preprocessing and genetic algorithm-optimized backpropagation neural networks (GA-BPNN) within 400–1100 nm [57]. Aboonajmi et al. developed PCA and radial basis function network (RBFN) models in the 300–1100 nm range to predict Haugh units (HU) and air cell height [58]. Dong et al. confirmed that pH prediction models for egg white using 550–850 nm spectra outperformed whole-egg models [59]. Sun et al. established a nonlinear support vector regression (SVR) model with locally linear embedding (LLE) for HU prediction (Rp = 0.8889), revealing inherent nonlinear relationships between spectral data and freshness-related chemicals [60]. Cruz et al. identified the equatorial region as optimal for HU modeling using portable NIR spectrometers [61], while Akowuah et al. achieved Rp values of 0.87 and 0.88 for refrigerated and ambient-stored eggs via partial least squares regression (PLSR) [62].

Despite advantages including rapid operation, minimal sample preparation, and structural preservation, Raman spectroscopy remains underutilized for egg freshness assessment [63]. Recent advances by Dong et al. identified optimal measurement zones: the top region for HU and albumen pH, averaged spectra for yolk index, and the bottom region for air cell parameters. Their PLSR models achieved correlation coefficients (0.862–0.977) with low root mean square errors, demonstrating high stability and accuracy [64].

Fluorescence spectroscopy is a sensitive, non-invasive technique that utilizes fluorescent compounds in eggs, including aromatic amino acids (tryptophan and tyrosine), vitamins (A and B2), and nucleotides [65]. Karoui correlated 672 nm fluorescence intensity (excited at 405–557 nm) to porphyrin derivatives, indicating higher signals in fresh eggs [66]. Romdhane et al. analyzed fluorescence signatures of Maillard reaction products (ex:360 nm/em:380–580 nm) and vitamin A (ex: 270–350 nm/em: 410 nm), achieving 97.7% prediction accuracy for vitamin A—a promising internal indicator for freshness assessment [67,68].

Hyperspectral imaging (HIS) integrates machine vision and spectral analysis to capture spatial–spectral data across ultraviolet to far-infrared ranges (λ/100 resolution) [69]. Yao et al. demonstrated its viability for freshness grading [70], while Dai et al. improved classification accuracy (0° light incidence) through ensemble learning [71]. Ma et al. identified feature wavelengths using genetic-PLS and developed least squares support vector machine (LS-SVM) models (Rp = 0.832) [72]. Çiftçi et al. enhanced transmission spectra through wavelet denoising and standardization, achieving PLSR correlations of 0.88–0.93 for HU [73]. Liu’s genetic algorithm-PLSR model achieved a calibration set correlation of 0.8118 [74]. Although HSI demonstrates significant potential for non-destructive quality control, current applications primarily focus on variety identification and freshness detection, with accuracy requiring enhancement.

**Table 5:** Chemometric methods in egg freshness detection via spectroscopy.

Method Type	Method	Performance	Reference
**Unsupervised**	Principal Component Analysis (PCA)	Prediction of Haugh units and egg air chamber height	[58]
**Supervised (Linear)**	Partial Least Squares Regression (PLSR)	Rp = 0.88–0.93, RMSE was low	[61,73]
**Supervised (Nonlinear)**	Support Vector Regression (SVR)	Rp = 0.8889	[60]
**Hybrid Optimization**	GA-BPNN (Genetic Algorithm-Backpropagation Neural Network)	Classification accuracy 94%	[57]
**Ensemble Learning**	LS-SVM (Least Squares Support Vector Machine)	Rp = 0.832	[72]

#### 2.5.3. Computer Vision Technology

Computer vision technology extracts, analyzes, and interprets visual information from image data through image processing methods [75]. During egg freshness decline, morphological changes occur in critical regions such as the air cell and yolk [76]. This technology utilizes algorithms and models to analyze egg images, extracting key visual features and correlating them with freshness labels, enabling precise and efficient freshness assessment. Current image processing methods based on computer vision comprise traditional approaches and deep learning methods [77].

Traditional image processing methods involve two primary stages: feature extraction and model construction. The feature extraction stage involves algorithmic extraction of freshness-related image features, converting them into numerical feature vectors [78]. The model construction stage develops machine learning models to establish associations between feature vectors and freshness labels for accurate evaluation [79]. Traditional methods have demonstrated significant effectiveness in freshness assessment. Wang et al. implemented color component extraction and binarization to isolate images from backgrounds, utilized gradient methods to identify air cells and calculate their area ratio to the entire egg, and constructed an SVM model for freshness discrimination, achieving 96.85% accuracy [80]. Sun et al. developed a Haugh unit prediction system that extracted shape features through threshold segmentation, histogram analysis, and morphological closing algorithms. Their multiple linear regression (MLR) model achieved an R^2^ of 0.87 and a root mean square error (RMSE) of 3.75, processing four eggs per second [81]. Qin et al. established correlations between air cell area ratios and Haugh units using color component extraction and threshold segmentation [82]. Nematinia et al. utilized egg image area indices as non-destructive weight parameters, confirming significant Pearson correlations with Haugh units, and achieved a maximum correlation coefficient of 0.93 using artificial neural networks (ANN) [83]. Narushin et al. developed a comprehensive non-destructive assessment system achieving 98.19% classification accuracy [84]. While these traditional methods offer viable solutions, their dependence on manual feature extraction constrains model accuracy and generalizability.

Deep learning methods have demonstrated remarkable progress in egg freshness assessment amid artificial intelligence advancements [85]. Egg freshness variations manifest as distinct image features detectable through machine vision [86]. Convolutional neural networks (CNN) excel at automatically learning rich feature representations from raw data, surpassing traditional methods in assessment accuracy and stability [87]. Researchers typically enhance performance by increasing model depth. Zhu developed a basic CNN model processing egg transillumination images, achieving 94.63% accuracy in three-level freshness classification [88]. Kim et al. created a CNN-long short-term memory (LSTM) model combining ResNet18 with LSTM networks for non-destructive Haugh unit prediction [89]. Fan et al. integrated machine vision with light transmittance data, achieving 92.5% recognition accuracy using BP neural networks [90]. Rho et al. developed a grading model using transmissive image features, achieving 99% and 98% accuracy in calibration and prediction sets [76]. Sun et al. proposed an online non-destructive method combining machine vision with dynamic weighing, achieving a prediction set correlation coefficient of 0.8653 [81]. Soltani et al. developed a multi-source data model integrating machine vision with dielectric constant detection [91,92].

Increasing network depth presents challenges through growing parameter counts and computational demands, impeding practical applications and rapid detection requirements. Consequently, research emphasis has shifted toward lightweight models for efficient evaluation. These models reduce parameters and complexity while maintaining accuracy through optimized architectures and parameter strategies, enabling deployment on resource-limited devices [93,94]. Models such as MobileNet, ShuffleNet, and EfficientNet have been successfully implemented in egg quality assessment, providing frameworks for lightweight freshness evaluation systems [95,96]. However, a machine vision’s limitation to physical characteristics, without chemical composition detection capability, restricts internal quality assessment compared to electronic nose or spectroscopic technologies.

### 2.6. Multi-Fusion Sensor Technology

Multi-sensor fusion is a technology that employs computational methods to automatically analyze and synthesize information and data from multiple sensors or sources under specific criteria, aiming to eliminate systemic uncertainties and provide accurate observational results and integrated intelligence [97]. It has been widely applied in military systems, industrial monitoring, intelligent inspection, robotics, image analysis, object detection and tracking, and automatic target recognition. In multi-sensor fusion detection systems, the architecture can be categorized into three hierarchical frameworks based on the level of information processing: data-level fusion (direct integration of raw sensor data), feature-level fusion (extraction and combination of salient features from preprocessed data), and decision-level fusion (aggregation of outputs from individual sensor-based decision models) [98].

The integration of advanced sensor technologies has revolutionized non-destructive egg freshness assessment by enabling multimodal data acquisition and analysis. Emerging trends emphasize sensor fusion to overcome limitations of single-modality approaches. For example, Liu et al. combined near-infrared (NIR) spectroscopy (950–1650 nm) with machine vision to assess both internal (yolk index) and external (shell cracks) quality parameters, achieving 96.5% accuracy via artificial neural networks (ANN) [99]. Similarly, Çiftçi et al. integrated transmission spectra (500–900 nm) and acoustic resonance analysis to predict air cell size and HU, improving PLSR model robustness (R^2^ = 0.89–0.93) through wavelet-based data fusion [100]. These hybrid systems leverage complementary data streams, enhancing prediction reliability under variable storage conditions. Despite progress, challenges persist in real-time deployment, including sensor miniaturization, cost-effectiveness, and algorithm interpretability. Future research should prioritize edge computing frameworks for on-farm applications and explainable AI models to bridge the gap between sensor outputs and biochemical mechanisms.

### 2.7. Artificial Intelligence (AI) Technology

The traditional egg deep-processing industry is undergoing an unprecedented technological revolution driven by advancements in artificial intelligence (AI). AI is permeating all aspects of egg processing—production, management, and sales—leveraging its unique advantages to enable transformative changes. In production, AI technologies like machine learning algorithms and data analytics are optimizing production line scheduling and operational efficiency. For instance, real-time data analysis enables predictive adjustments to production plans, ensuring alignment with market demands while minimizing waste [101,102]. AI-powered hyperspectral imaging (HSI) further enhances defect detection (e.g., cracks and stains) and freshness grading by analyzing spectral features correlated with Haugh units [103]. In management, AI-driven platforms integrate IoT sensors and cloud computing to monitor environmental parameters (temperature and humidity) and equipment performance in real time, reducing operational risks and costs. For example, XGBoost models predict egg production rates and weights with high precision (MAPE <7.03% for production rate; <1.1% for weight) by analyzing age, feed intake, and environmental factors [104]. These innovations not only stabilize product quality but also lay the foundation for scalable, sustainable production systems.

### 2.8. Other Detection Technologies

To facilitate a comparative analysis of various techniques, this study has compiled the primary methods for assessing egg freshness (Table 6). Beyond the previously discussed techniques, researchers have investigated additional non-destructive methods for determining egg freshness. Volgyi implemented microwave sensors to evaluate egg freshness, documenting changes in microwave attenuation and the effective radar cross-section of egg transmission and reception during a 30-day storage period [105]. Zhang and colleagues utilized a weak luminescence imaging detection system to examine the luminescence characteristics of eggs during storage [106]. Their research revealed that luminescence intensity peaked on the third day followed by oscillatory decay, demonstrating that ultra-weak luminescence intensity varies among eggs and correlates with their vitality; eggs with greater vitality emit stronger light. This approach offers simplicity, non-invasiveness, and high sensitivity. Fadchar developed a computer vision system integrated with Matlab software (R2024a) to obtain egg color parameters [107]. Through experimental analysis, they determined the half-value of eggs, utilizing this data to construct a BP neural network model that identified optimal relationships between egg freshness and image color parameters.

### 2.9. Instrumental Analysis Techniques

Egg quality, influenced by factors such as the hen’s diet, age, health, and production/storage conditions, is now objectively assessed through advanced instrumental methods that provide precise, reliable measurements of physical, chemical, and microbiological properties [108,109]. Spectrophotometry, for instance, quantifies eggshell color, opacity, and thickness by analyzing light transmission or reflection, while electrical conductivity (EC) testing evaluates freshness by measuring ion movement through the shell, with fresher eggs exhibiting higher EC due to intact permeability. Ultrasound analysis generates detailed images of internal structures, such as yolk integrity, albumen consistency, and air cell size, enabling non-destructive quality evaluation. Automated inspection systems combine high-resolution cameras and image-processing algorithms to detect surface defects (e.g., cracks and shape irregularities) and classify eggs based on visual standards. Mass spectrometry offers proteomic insights by identifying changes in egg white protein expression, linking molecular profiles to nutritional and functional quality. Microbiological testing, including bacterial culture and surface swabbing, ensures food safety by detecting pathogens like Salmonella spp. These techniques—spanning physical, chemical, and biological analyses—collectively enhance accuracy in quality control, streamline production processes, and safeguard consumer trust in egg products by addressing both visible and hidden quality parameters.

## 3. Health Risks of Poor-Quality Eggs

Due to constraints in funding, technology, and management, some domestic egg producers prioritize profit maximization by using large amounts of cheap, low-quality feed. To enhance visual appeal and attract consumers, they illegally employ prohibited chemical substances. Additionally, antibiotics are excessively used to mitigate disease risks. These practices not only lead to nutritional imbalances and degraded egg quality but also result in excessive drug residues and contamination by toxic substances. Furthermore, backward poultry farming environments and poor management contribute to egg pollution through waste, sewage, and exhaust emissions containing harmful heavy metals and microorganisms, posing potential health hazards.

Microbiological hazards remain the most significant threat to food safety and the primary cause of foodborne illnesses [110]. Moreover, lax production controls and incomplete microbial sterilization in some facilities leave eggs contaminated with pathogens or allow microbial proliferation during processing, storage, or transportation. Common microorganisms in eggs include bacteria and fungi. Bacterial contaminants include Staphylococcus, Streptococcus, Escherichia coli, Proteus, Pseudomonas, Bacillus, and Salmonella, while fungal contaminants include Aspergillus, Penicillium, and Mucor [111]. Eggs carrying these microorganisms can cause diseases and food poisoning in humans, weakening immunity. Studies indicate that approximately 50% of fresh eggs carry pathogenic bacteria, molds, or parasite eggs, with Salmonella being the predominant pathogen [112]. Salmonella comprises numerous strains, some pathogenic to humans, others to animals, and some to both. Salmonella infections in humans, transmitted primarily via the digestive tract, manifest as typhoid, paratyphoid, food poisoning, and septicemia.

## 4. Literature Search Methodology

This review employed a systematic approach to identify, select, and analyze relevant studies on egg freshness evaluation methods.

### 4.1. Search Strategy

Primary scientific databases, including Web of Science, PubMed, Scopus, and Google Scholar, were queried using keywords such as the following: “egg freshness evaluation”, “non-destructive detection of egg quality”, “Haugh unit measurement”, “spectroscopic analysis of eggs”, and “machine learning in egg quality assessment”.

### 4.2. Synthesis Approach

A thematic analysis categorized methods into conventional (sensory and physicochemical) and emerging (AI and multi-sensor fusion) techniques. Comparative tables (Table 3, Table 4, Table 5 and Table 6) were developed to highlight performance metrics across studies.

## 5. Conclusions and Future Prospects

Eggs remain a cornerstone of global nutrition, yet their perishable nature presents critical challenges for quality assurance across supply chains. Conventional freshness assessment methods, including Haugh units and yolk indices, face fundamental limitations in objectivity, efficiency, and scalability. While sensory evaluations are inherently subjective, destructive physicochemical analyses preclude real-time monitoring. Emerging non-destructive technologies demonstrate complementary capabilities: electronic nose systems effectively profile volatile organic compounds indicative of biochemical degradation; spectroscopic modalities enable molecular-level tracking of protein denaturation and lipid oxidation; and computer vision excels in detecting structural defects like microcracks. Nevertheless, current single-mode detection paradigms inadequately address the multidimensional deterioration mechanisms involving synergistic physical, enzymatic, and microbial interactions.

Looking ahead, systematic integration of multi-sensor data streams through machine learning architectures will prove transformative. Hybrid models combining hyperspectral imaging, dielectric spectroscopy, and triboelectric nanogenerators could generate comprehensive quality fingerprints. The development of handheld multimodal sensors—incorporating microfluidic preconcentrators for volatile enrichment and miniaturized NIR spectrometers—would enable field-deployable solutions for real-time supply chain diagnostics. This integration will ultimately enable concurrent monitoring of physical integrity, biochemical stability, and nutritional preservation, overcoming the critical “single-mode detection” bottleneck that currently constrains industrial applications.

## Figures and Tables

**Table 1:** Comparison of Haugh unit grading standards.

Freshness Grade	Haugh Unit Value		
	USDA	MOA	MOC
AA	>72	>72	>72
A	60–72	60–72	>60
B	<60	31–59	>55
C	/	<31	/

**Table 2:** Comparison of yolk index grading standards.

Freshness Grade	Yolk Index	Storage Time
AA	>0.35	<7 day
A	0.25–0.35	8–15 day
B	<0.25	>15 day

**Table 3:** Comparison of egg freshness indicators in traditional detection methods (in China).

Indicator	China National Standards	Detection Mode	Detection Method	Measurement Convenience	Correspondence with Freshness	Measurement Accuracy
Sensory Index	Yes	Destructive	Physical	Fast	Average	Poor
Air Cell Height	Yes	Non-destructive	Physical	Average	Close	Accurate
Haugh Unit	Yes	Destructive	Physical	Average	Close	Accurate
pH Value	No	Destructive	Physical	Average	Close	Accurate
Yolk Index	No	Destructive	Physical	Average	Close	Accurate
Total Volatile Basic Nitrogen	No	Destructive	Chemical	Slow	Close	Accurate

**Table 4:** Comparison of egg freshness indicators across countries.

Indicator	China National Standards	USA (USDA/FDA)	EU (EC)	Japan (JIS)
Sensory Index	Yes (GB2748)	Yes (appearance, odor)	Yes (shell cleanliness)	Yes (shell disinfection)
Air Cell Height	≤8 mm	Non-destructive testing	≤0.8 cm	Stability-focused
Haugh Unit	≥60 (Grade B)	≥72 (AA Grade)	≥60	≥70
pH Value	Not included	Not included	Used for spoilage detection	Correlated with TVB-N
Yolk Index	Not included	≥0.40 (AA Grade)	≥0.35	≥0.38
Total Volatile Basic Nitrogen	Not included	Used for spoilage detection	≤10 mg/100 g	Required (JIS Z 8401)

**Table 6:** Comprehensive comparison of egg freshness testing methods.

Detection Methods	Principle	Detection Indicators	Operating Steps	Advantage	Disadvantage	Application Scenarios	Accuracy	Cost	Destructive	Reference
Sensory Detection	Judging appearance, smell, and shaking sound through visual, auditory, and tactile senses.	Eggshell integrity, yolk condition, off-odors, and shaking sound (air cell size).	Direct observation of eggshell cracks, shaking to listen for sounds, light transmission to view the air cell, and water float test.	No equipment needed, simple to operate, suitable for household or small-scale use.	Highly subjective, experience-dependent, low accuracy.	Suitable for households, small-scale production, or preliminary screening.	low	low	no	[16,17,18,19,20,21]
Physical Detection	Measuring physical parameters such as weight, air cell size, and shell strength.	Weight loss rate, air cell height, and eggshell thickness.	Weighing, air cell measuring device, and eggshell strength testing machine.	Low cost and relatively simple to operate.	Requires manual operation, with some indicators (such as air cell) needing to be measured by breaking the shell, resulting in lower efficiency.	Auxiliary testing in laboratories or small-scale production.	medium	low	partial destruction	[22,23,24]
Chemical Detection	Detecting changes in the internal composition of eggs, such as pH value and volatile basic nitrogen.	Yolk pH, protein Haugh units, and volatile basic nitrogen (TVBN).	Cracking the egg and using a pH meter, texture analyzer, or chemical reagents for analysis.	Results are objective, highly accurate, and quantifiable.	Requires sample destruction, time-consuming, and necessitates specialized equipment and personnel.	Laboratory research or high-precision testing requirements.	high	mid-to-high	destructive	[25,26,27,28,29,30,31,32,33,34,35,36,37,38]
Electronic Nose	The sensor array captures the volatile gases emitted by eggs and analyzes their odor characteristics.	Volatile organic compounds (e.g., hydrogen sulfide, ammonia).	Placing the egg in a closed container, where an electronic nose collects gas signals for modeling and analysis.	Non-destructive, rapid, and can be automated.	Affected by environmental temperature and humidity, requiring regular calibration, and the equipment cost is relatively high.	Batch testing in production lines or storage environments.	mid-to-high	high	no	[42,43,44,45,46,47,48,49,50,51,52,53]
Spectral Analysis Technology	Near-infrared/Raman spectroscopy analysis of egg components (moisture, protein, etc.).	Moisture content and changes in protein structure.	Using a spectrometer to scan the eggshell or egg liquid, combined with chemometric models for analysis.	Non-destructive, capable of detecting internal components, and highly accurate.	Equipment is expensive, requires complex modeling, and has high demands on the operator’s skills.	Laboratory or high-end quality control scenarios.	high	high	no	[54,55,56,57,58,59,60,61,62,63,64,65,66,67,68,69,70,71,72,73,74]
Computer Vision Technology	Image processing technology analyzes eggshell color, air cell size, yolk shape, and more.	Eggshell texture, air cell area, and yolk profile.	Using a high-resolution camera to capture images, with image algorithms extracting features and classifying them.	Non-destructive, can be integrated into automated production lines, and highly efficient.	Dependent on lighting conditions, requiring high image quality, and the initial modeling cost is high.	Automated sorting in large-scale production.	mid-to-high	mid-to-high	no	[75,76,77,78,79,80,81,82,83,84,85,86,87,88,89,90,91,92,93,94,95,96]
Integrated Model	Fusion of multiple technologies (e.g., electronic nose + spectroscopy + machine learning) for multi-dimensional analysis.	Multi-parameter combined indicators such as odor, composition, and appearance.	Multi-sensor data collection, with comprehensive evaluation using machine learning models such as SVM and neural networks.	Extremely high accuracy, capable of comprehensive assessment of freshness.	The system is complex, with extremely high costs, and requires a large amount of data to train the model.	High-end research or high-precision industrial testing (such as export quality certification).	extremely high	extremely high	no	[97,98,99,100]

## Data Availability

No new data were created or analyzed in this study.
